# Learnable digital signal processing: a new benchmark of linearity compensation for optical fiber communications

**DOI:** 10.1038/s41377-024-01556-5

**Published:** 2024-08-13

**Authors:** Zekun Niu, Hang Yang, Lyu Li, Minghui Shi, Guozhi Xu, Weisheng Hu, Lilin Yi

**Affiliations:** State Key Lab of Advanced Optical Communication Systems and Networks, School of Electronic Information and Electrical Engineering, Shanghai Jiao Tong University, Shanghai, 200240 PR China

**Keywords:** Fibre optics and optical communications, Optical physics

## Abstract

The surge in interest regarding the next generation of optical fiber transmission has stimulated the development of digital signal processing (DSP) schemes that are highly cost-effective with both high performance and low complexity. As benchmarks for nonlinear compensation methods, however, traditional DSP designed with block-by-block modules for linear compensations, could exhibit residual linear effects after compensation, limiting the nonlinear compensation performance. Here we propose a high-efficient design thought for DSP based on the learnable perspectivity, called learnable DSP (LDSP). LDSP reuses the traditional DSP modules, regarding the whole DSP as a deep learning framework and optimizing the DSP parameters adaptively based on backpropagation algorithm from a global scale. This method not only establishes new standards in linear DSP performance but also serves as a critical benchmark for nonlinear DSP designs. In comparison to traditional DSP with hyperparameter optimization, a notable enhancement of approximately 1.21 dB in the Q factor for 400 Gb/s signal after 1600 km fiber transmission is experimentally demonstrated by combining LDSP and perturbation-based nonlinear compensation algorithm. Benefiting from the learnable model, LDSP can learn the best configuration adaptively with low complexity, reducing dependence on initial parameters. The proposed approach implements a symbol-rate DSP with a small bit error rate (BER) cost in exchange for a 48% complexity reduction compared to the conventional 2 samples/symbol processing. We believe that LDSP represents a new and highly efficient paradigm for DSP design, which is poised to attract considerable attention across various domains of optical communications.

## Graphical abstract

**Figure:**
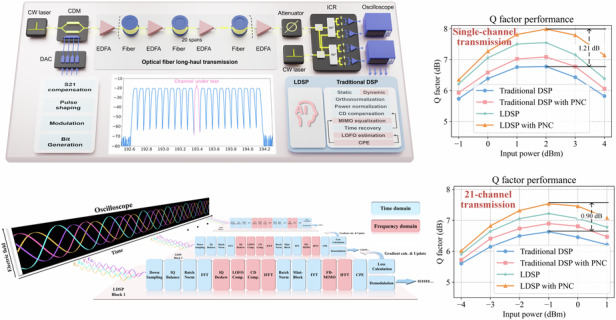
A learnable DSP (LDSP) is introduced as a new benchmark for linear compensation in optical communications, which optimizes DSP blocks globally by leveraging the power of learnable perspectivity.

## Introduction

With the advancement of modern information technology, optical fiber communications have been upgraded in various aspects—from signal processing to fiber manufacturing—to enhance transmission capacity in recent years^1–5^. Digital signal processing (DSP), a mature technology adept at compensating for various linear effects, is essential for optical fiber transmission systems^6–11^. Deriving from physical models, the well-established DSP structure prioritizes low complexity and high performance^12–14^. In addition, efforts to achieve higher capacity are often limited by nonlinearities, leading to a reliance on nonlinear compensations (NLC)^15,16^. Common NLC techniques, such as perturbation compensations, are typically benchmarked against linear DSP^17–21^. It is essential that enhancements in nonlinear performance are evaluated against a baseline where linear impairments have been effectively minimized. Failing to do so may result in a biased estimation of performance, influenced by the benchmark of the linear compensation. Thus, the design of linear DSP must be approached with high performance and low complexity, aiming to address linear effects comprehensively.

Typically, conventional DSP operates block-by-block, with each block addressing a specific task. For instance, static and dynamic equalizers are designed to counteract chromatic dispersion (CD) and polarization mode dispersion (PMD) in the fiber link, respectively^8^. However, these equalizers may exhibit limited performance given the comprehensive and global nature of fiber link effects, due to a lack of global cooperation. Besides, reducing the complexity of existing DSP architectures with low processing rate while maintaining exceptional performance remains a challenge. The utilization of deep learning (DL) models offers the advantage of a data-driven approach, enabling signal processing without the need for expert knowledge^22–25^. Recent research has proposed learned models to address fiber nonlinearities^24–27^. Notably, DL techniques have been applied to digital backpropagation (DBP) by regarding the iterative linear and nonlinear operations of DBP as the equivalent operations of a deep neural network (DNN). This approach, known as learned DBP (LDBP), leverages DL techniques, demonstrating through simulation and experiment how machine learning-based approaches can reduce complexity while achieving comparable performance^20,21^. The key technology of deep learning models is gradient descent through backpropagation algorithm. However, the traditional DSP framework is incompatible with DL techniques due to its reliance on model-driven solutions. While some works have embedded DL algorithms within DSP, the DL and DSP blocks remain separate entities, where the focus of optimizations primarily centered on the DL algorithms^22,28^. Furthermore, the DL is a static compensation while DSP is an adaptive compensation. Redesigning the entire DSP framework to incorporate DL methods in pursuit of performance enhancements may not be cost-effective. Therefore, it would be appealing to achieve performance improvements using DL techniques without requiring significant changes to the existing DSP framework.

Here, we propose a DSP scheme that we call learnable DSP (LDSP), reusing the traditional DSP framework while introducing the DL optimization framework. LDSP treats the entire DSP as a learnable structure and globally optimizes the DSP blocks. Specifically, LDSP reuses the traditional DSP model, with the distinction that all DSP blocks are made differentiable and learnable. These learnable parameters can be optimized using stochastic gradient descent (SGD)^29^ and its variants through the backpropagation algorithm, such as the Adam algorithm^30^, which adaptively modifies the learning rate. In this way, all LDSP blocks share processing information globally, enabling comprehensive utilization of DSP resources for linear compensation. Technically, LDSP is a traditional DSP framework with online training, harnessing the combination of expert knowledge and the advantages of DL. As demonstrated in the subsequent analysis, one LDSP module can achieve several different functions, showing the high efficiency. The frequency compensation block in LDSP can simultaneously compensate the IQ skew, local oscillator frequency offset (LOFO), and CD. Moreover, the sampling errors can be detected through the IQ skew compensation and time recovery (TR) can be removed, saving the DSP resources. The power normalization can align the modulation map and address the clock tone leakage of digital to analog converter (DAC).

In comparison to traditional DSP with hyperparameter optimization, after conducting 1600 km fiber experimental transmission for a 400 Gb/s signal, LDSP exhibits a noteworthy 0.77 dB and 0.58 dB Q factor improvement for single-channel and 21-channel transmission, respectively. Combined with NLC, there is an observed performance enhancement, achieving a maximum gain of 1.21 dB and 0.9 dB. The implementation of the LDSP architecture facilitates communication between DSP modules through backpropagation algorithm, enabling comprehensive compensation for global signal impairments and thereby enhancing DSP efficiency. One noteworthy advantage of LDSP is its ability to autonomously learn various DSP configurations, minimizing the requirement for fine manual tuning of the DSP modules. Additionally, the need for a separate TR algorithm can be eliminated within the LDSP framework. Besides, the symbol-rate processing can be achieved with little bit error rate (BER) cost. In this way, LDSP not only achieves performance improvements but also demonstrates a remarkable 48% reduction in complexity compared to 2 samples/symbol processing in the typical system. The LDSP scheme, being an online data-driven model, is compatible with DL methods, thus ensuring compatibility and leveraging their benefits. Attaining optimized performance in linear DSPs is crucial for accurately assessing the advantages offered by nonlinear DSPs, seeking for higher capacity. It is worth noting that the methods discussed in this study hold relevance to the broader field of DSP, which has been gaining considerable attention in various optical fiber communication scenarios, including short-reach, medium-reach, and long-haul links.

## Results

### Learnable DSP

**Fig. 1:**
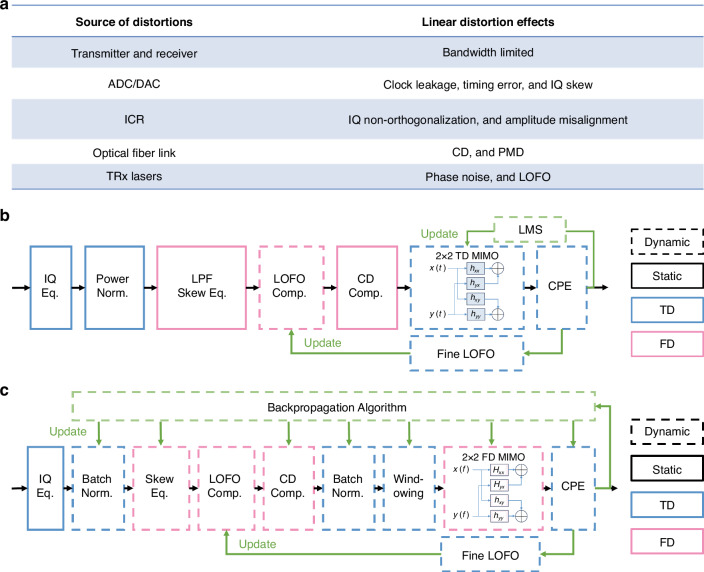
**LDSP scheme details.** **a** The linear distortions in the optical fiber long-haul transmission system, including the transmitter, channel, and receiver. **b** The traditional DSP structure. **c** The LDSP structure

In coherent optical fiber long-haul transmission systems, various linear distortions, depicted in Fig. 1a, arise due to imperfections in subsystems. Here, we focus on linear effects, thoroughly explored in conventional DSP algorithms. The system complexity stems from the interaction of linear impairments with different components, demanding precise design and DSP in a strategically structured sequence. DSP plays a pivotal role in identifying and addressing these mixed effects sequentially, critical for maintaining system integrity and performance. Traditional DSP modules, differentiated into static and dynamic equalizations to counter distinct system distortions (see Fig. 1b), address specific distortion characteristics. Static equalizations, such as CD compensation (CDC), are designed to address distortions that remain constant over time for a fixed optical transmission link. On the other hand, dynamic equalizations, such as those for PMD and phase noises, target distortions that vary dynamically during transmission. For PMD, a time-domain multiple-input multiple-output (TD MIMO) equalizer is utilized with 2 samples/symbol processing, while carrier phase estimation (CPE) handles phase noise. The CPE process leverages a blind-phase search (BPS) algorithm for adaptive phase noise tracking. Subsequently, the least mean squares (LMS) algorithm updates MIMO parameters, ensuring the optimal performance. Additionally, the LOFO may exhibit slow variations over time, necessitating the implementation of a fine LOFO estimation process, which is then feedbacked to enhance the accuracy of the LOFO estimator. By categorizing the DSP modules in this manner, the system can effectively address the specific characteristics of each distortion type. However, as each module is optimized locally to mitigate a particular effect, it may lead to an underutilization of DSP functions and result in local optimization. To achieve enhanced performance, a potential way involves increasing the complexity of DSP. For instance, the power normalization module ensures the correct signal amplitude but does not encompass clock leakage compensation, which necessitates a separate DSP module.

In this work, the proposed LDSP is a learning-based DSP framework as depicted in Fig. 1c, that extensively leverages existing DSP modules, such as IQ skew, CD compensation, CPE, LOFO estimator, and others. While the majority of the DSP modules remain the same, each DSP module is treated as a linear layer of a deep neural network (DNN), and its parameters are optimized using a learning algorithm through backpropagation, specifically the SGD method. This approach enables performance optimization from a global perspective, offering a more holistic and effective solution.

The LDSP scheme optimizes the IQ skew, LOFO compensation, and CD compensation within the same frequency-domain module. All the parameters of the DSP modules are set to be learnable, which means they can be adjusted and optimized adaptively during the signal processing. To avoid the IQ signal mixture after CD compensation, the IQ de-skew is performed before the CD compensation in the frequency-domain. Prior to the MIMO compensation, the incoming signals are divided into several mini blocks to address dynamic effects such as PMD and phase noises. Each signal block undergoes windowing through a trainable time-domain filter, a strategy designed to effectively counteract spectrum leakage. The frequency-domain MIMO (FD MIMO) compensation is performed using the overlap-and-save method, supporting processing at a rate of 1.0–2.0 samples per symbol^31^. A BPS algorithm is used to perform a learnable CPE. By employing a differential approach, the LDSP scheme can adjust the BPS parameters to track the phase noise according to the channel distortions present. This allows for robust and efficient CPE in the LDSP framework.

In addition to reusing existing DSP modules, the LDSP framework also seamlessly incorporates the classical DL structure, capitalizing on its learning capabilities. Classical DL structures encompass a wide range of effective algorithms, such as batch normalization (BN) layers, residual layers, and adaptive optimizers. Here, we introduce a novel modification to the power normalization module by integrating a complex BN algorithm, drawing inspiration from the BN scheme^32^. Unlike traditional power normalization, complex BN not only aligns the amplitude but also incorporates a bias addition operation. The mathematical equation depicting this modification is presented below:

1 $$y=\gamma \frac{(x-{\mu }_{x})}{\sqrt{{p}_{x}}}+\beta$$

where ${\mu }_{x}$ and ${p}_{x}$ are mean and power of signal *x*. The *γ* and *β* are the learning parameters. Note that the bias *β* typically represents a numerical value used in conventional BN algorithms. In contrast to the typical BN algorithm, the bias *β* is a complex sequence with zero mean, equally length as signal *x*. The incorporation of a bias sequence instead of a single complex value is driven by two primary considerations: (1) It stabilizes adaptive training during the Constant Modulus Algorithm (CMA) and Multi-Modulus Algorithm (MMA) phases. Utilizing a single bias value often results in overfitting to the modulus of the CMA or MMA. (2) It compensates foradditive distortions such as clock leakage from non-ideal DAC, where clock information inadvertently embedded in the signal can lead to symbol interference. We find the bias sequence can be utilized for clock leakage compensation, enhancing the overall performance of LDSP. The complex BN modules have the ability to automatically adjust power and bias based on the signal characteristics.

In term of the optimizer, the LDSP uses the SGD with momentum algorithm. This optimizer, different from the basic SGD, incorporates momentum to achieve faster convergence and better performance, which is widely used for training modern DL architectures. One could also use the adaptive variants, such as Adam. Adaptive learning rate methods are theoretically better suited to this task, as they can control their updates in response to the signal’s changing nature. While Adam offers superior convergence properties in many cases, they are computationally more intensive than simple SGD or momentum-based methods. This might be a consideration if the model needs to be deployed in real-time signal processing applications.

### Schematic illustration of the LDSP in the experiment

**Fig. 2:**
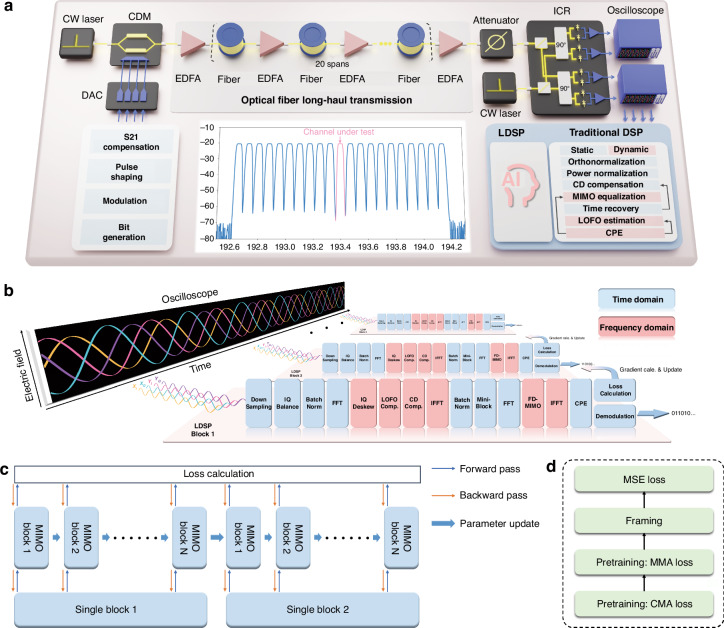
**Schematic illustration of the LDSP in the experiments.** **a** Schematic of the experimental optical fiber long-haul transmission system, including the transmitter, channel, and receiver. **b** The blocked data processing with LDSP. The received signals, sampling from the oscilloscope, is conducted block-by-block with the same LDSP structure. **c** The structure of the forward pass, backward pass, and parameter update within LDSP. **d** The employed loss function during the training and tracking procedure

Experiments are conducted to determine the effectiveness of LDSP in practice. The experimental setup for a 1600 km optical fiber transmission is depicted in Fig. 2a, with comprehensive details provided in the Materials and Methods section. To process a long-time signal series efficiently, the signals are divided into several signal blocks, shown in Fig. 2b. Signals are processed and the LDSP framework is updated using the learning algorithm in a time-sequential manner. This approach allows for more manageable and computationally efficient processing of the entire signal series while maintaining the LDSP performance through iterative learning updates. The optimization task is supported by the Pytorch framework^33^, which the version is 2.0 in this work. The received signal is first resampled to 1.0–2.0 samples/symbol and then performed DSP. In the following, we will provide the detail structures of LDSP module, showing how to improve the efficiency and perform learning algorithms.

It is noteworthy that all DSP operations must be implemented differentially and then the backpropagation algorithm can be performed normally. After passing through the entire LDSP module, the loss is computed based on the loss function, and gradients are calculated using the error backpropagation algorithm. The overall process, including the forward path, backward path, and parameter updates, is illustrated in Fig. 2c. The training process for gradient calculation includes two steps: (1) During the mini-block operation, the loss and gradients are promptly calculated, and the parameters are updated for the subsequent mini-block signals. Additionally, the gradients of whole block parameters, such as CD and IQ skew parameters, are accumulated and divided by the mini-block number, although the parameters remain fixed during this step. (2) Updating the parameters of large blocks according to the averaged gradients. This method allows for averaging out noisy gradients, thereby stabilizing the training process. LDSP then proceeds to the next signal block. By allowing the parameters to be learned, the LDSP scheme can adapt and optimize the DSP operations based on the specific characteristics of the optical fiber transmission system. Moreover, the gradient backward process functions as an internal interconnection element. Consequently, LDSP enhances the overall performance and efficiency of the DSP system, allowing for improved signal processing and mitigating various distortions in a more integrated and coordinated manner.

The LDSP is executed in three sequential steps, employing distinct loss functions as depicted in Fig. 2d. Initially, due to the inability to perform framing, the learnable parameters are trained in a blind manner. At the beginning of the processing, the loss function is set as the CMA. To address the singularity issue encountered in CMA training, we employ orthogonal training, wherein solely the X branch taps undergo training while the Y branch taps are arranged orthogonally to the X branch taps^34^. Following several iterations, the MIMO taps can transition to full training. Although convergence may not be attained through orthogonal training, it serves to mitigate the singularity problem, facilitating subsequent pretraining process. Once CMA convergence is achieved, the loss function switches to the MMA^35^. Simultaneously, framing is performed. Upon successful framing, the LDSP transitions to the data-aided tracking case, where the loss function becomes the mean-square error (MSE) loss function:

2 $${L}_{\rm{MSE}}=\frac{1}{N}\mathop{\sum }\limits_{i=1}^{N}\Vert {y}_{i}-{\hat{y}}_{i}\Vert$$

where *N* represents the number of symbols in a mini block, ${y}_{i}$ is the label symbol, and ${\hat{y}}_{i}$ is the processed symbol from the LDSP. MSE loss provides a mathematically tractable expression that is particularly sensitive to large errors, leading to a second-order dependence of the cost function on the unknown coefficients^36^. Consequently, MSE loss is widely utilized in various statistical and machine learning contexts to evaluate performance^14–21^.

The above overview provides an introduction to the LDSP scheme, covering its conceptual framework, structural components, and learning procedure. LDSP treats the entire DSP modules as a learnable model, harnessing the benefits of a learnable framework to facilitate global optimization through the interconnections within the DSP modules. Furthermore, the reuse of the same structure allows a single DSP module to effectively process multiple channel distortions, maximizing the utilization of DSP resources, which will be shown in the next section. The subsequent section presents the experimental results of the proposed LDSP.

### Performance gain of learnable DSP

To demonstrate the performance improvement achieved by the LDSP scheme, a comparison was made between LDSP and traditional DSP in terms of Q factor performance, which can be calculated from the bit error rate (BER) using Eq. (3):

3 $$Q=20{\log }_{10}\left(\right.\sqrt{2}erf{c}^{-1}(2{\rm{BER}})$$

The traditional DSP followed the same processing procedure as LDSP. The MIMO update transitioned from CMA to MMA, and finally to data-aided least mean square (LMS). Both LDSP and traditional DSP omitted the time recovery module. The MIMO configuration used classical 2 × 2 taps. The traditional DSP had a sampling rate of 2.0 samples per symbol which is the typical case, whereas LDSP operated at a rate of 1.25 samples per symbol. In the LDSP framework, the signal block consisted of 512 symbols divided into 8 mini blocks, with each mini-block processing 64 symbols simultaneously. Both single-channel and 21-channel transmission has the same hyperparameter settings. The learning rate (LR) for CMA and MMA updates was set to 1.0e−3 for coarse training, while the MSE update utilized a LR of 1.0e-4 for fine tracking. The selection of LR was based on the processing step, where larger LR values aided the convergence of pretraining in CMA and MMA updates. During the tracking process, as most static channel effects had converged, the LR could be reduced to fine-tune the static effects and track the dynamic effects.

**Fig. 3:**
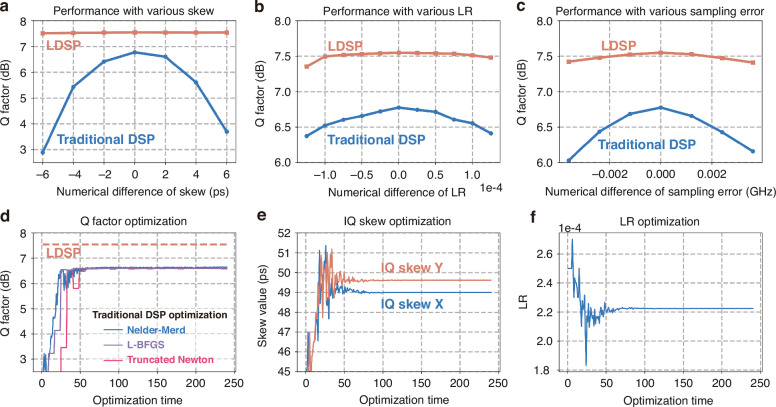
**The performance on hyperparameters for traditional DSP and LDSP.** **a**–**c** Q factor performance for traditional DSP and LDSP as functions of skew, LR and sampling errors. **d** The optimization procedures on traditional DSP with the Nelder-Mead, L-BFGS, and Truncated Newton method. **e**, **f** IQ skew and LR of traditional DSP during the Nelder-Mead optimization

It should be noted that hyperparameters can impact performance, and an optimization procedure is necessary in practice. Both traditional and learnable DSP require such optimization, and LDSP can automatically optimize some parameters, such as taps of CDC, IQ skew values and so on. This study involves parameter optimization, with results illustrated in Fig. 3, conducted on a single-channel transmission at 2.0 dBm over 1600 km. In practice, the IQ skew, LR and sampling error significantly affect performance. Figure 3a–c demonstrates performance variations as functions of IQ skew, LR, and sampling error, respectively. Parameters are individually searched, holding others at optimal levels. Traditional DSP showed sensitivity to these parameters, particularly IQ skew, with potential losses exceeding 4 dB in the worst scenarios. In contrast, LDSP’s performance remained relatively stable, which allows for autonomous adaptation to various configurations, reducing the need for extensive manual DSP module tuning.

In practice, the hyperparameters of DSP, such as IQ skew, MIMO learning rate, typically require manual adjustment based on final DSP performance. This process can be laborious and time-consuming, particularly when numerous hyperparameters are involved. To enhance the efficiency of optimization, hyperparameter optimization algorithms for DSP could be employed to determine the optimal performance^37–39^. The optimization procedure is executed in five steps: (1) construct the set of hyperparameters to be optimized; (2) determine the initial guesses for these hyperparameters; (3) apply these guesses within the DSP to assess performance; (4) use the optimization algorithm to update the hyperparameters based on observed performance; (5) repeat from step (3) until the performance converges. This method allows for efficient optimization and determination of DSP hyperparameters. Here for fairness in comparison, traditional DSP hyperparameters are optimized together using the standard Nelder-Mead algorithm in this paper. The Nelder-Mead algorithm is a popular optimization technique used for minimizing a function in a multidimensional space^37^. It’s particularly useful for hyperparameter optimization where the function to be minimized is not differentiable. We have also tested this optimization problem using other methods, specifically the Truncated Newton (TNC) and L-BFGS methods. The DSP optimizations focus on the IQ skew, sampling error, and LR, while other hyperparameters for DSP and LDSP, such as the MIMO size, are set to be consistent. Figure 3d demonstrates the optimization procedure, with LDSP’s performance at 7.55 dB serving as a baseline (dashed line). Trajectories of IQ skew and LR are shown in Fig. 3e, f. The traditional DSP optimization results, as depicted in Fig. 3d, demonstrate that all three optimization methods achieve similar performance, converging to approximately 6.77 dB. These results highlight LDSP outperforming, which achieves enhanced performance through global optimization, rather than solely optimizing parameters within some DSP modules.

**Fig. 4:**
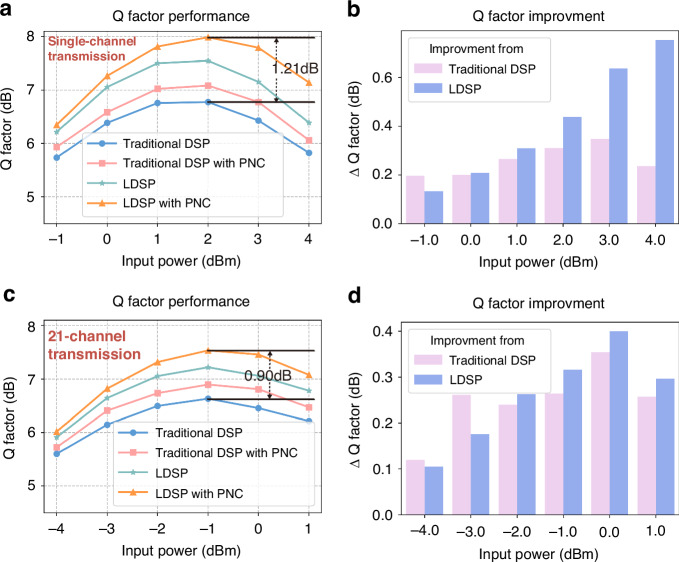
**The performance comparison of LDSP scheme and traditional DSP.** **a** 50-GBaud 16-QAM Q factor performance as a function of input power after 1600-km fiber single-channel transmission. **b** The bar chart of Q factor improvement using PNC after 1600-km fiber single-channel transmission. **c** 50-GBaud 16-QAM Q factor performance as a function of input power after 1600-km fiber 21-channel transmission. **d** The bar chart of Q factor improvement using PNC after 1600-km fiber 21-channel transmission

The experimental results revealed that LDSP achieved a significant improvement in Q factor performance compared to traditional DSP after parameters optimization, with a gain of 0.77 dB at 2.0 dBm input power for single-channel 1600 km transmission, as illustrated in Fig. 4a. Regarding the 21-channel WDM transmission over 1600 km, as illustrated in Fig. 4c, there is an observed performance enhancement, achieving a maximum gain of 0.56 dB. LDSP consistently outperformed traditional DSP across all input power levels.

To demonstrate the efficacy of the proposed LDSP under NLC, we employed the perturbation nonlinear compensation (PNC) algorithm^17–19,40^. The results revealed that LDSP-enhanced PNC exhibits notable NLC capabilities due to the increased accuracy in linear compensation, enhancing the precision of nonlinear estimation. This improvement is quantitatively evident, with performance gains of 1.21 dB and 0.9 dB in single-channel and 21-channel transmission, respectively. Moreover, as illustrated in Fig. 4b, d, we compared the PNC gains under conventional DSP and LDSP. Essentially, as power increases, both the optical signal-to-noise ratio (OSNR) and nonlinear effects are simultaneously enhanced. Consequently, the Q factor initially improves after PNC, due to enhanced OSNR and nonlinearities. However, as nonlinear effects become dominant, the improvement may deteriorate, constrained by the limited efficacy of PNC. In conventional DSP, the residual uncompensated linear effects add additional noise to the NLC algorithm, thereby limiting its effectiveness as power increases. Conversely, the nonlinear gain based on LDSP progressively amplifies, stemming from more accurate nonlinear estimations. Our findings suggest that NLC performance can be enhanced using LDSP, particularly in scenarios with apparent nonlinear effects. These results highlight LDSP’s potential as a novel benchmark, significantly aiding in the performance assessment of nonlinear algorithms.

### Learned parameters analysis

**Fig. 5:**
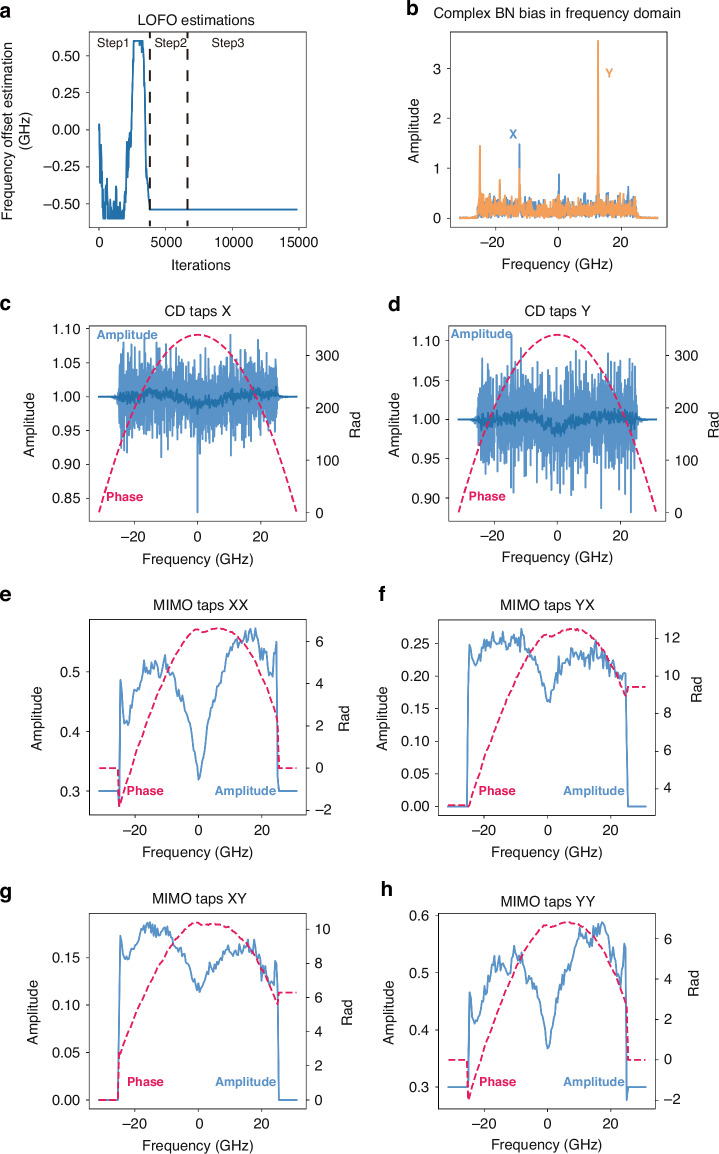
**The learned parameters after learning process.** **a** The LOFO estimation during the learning procedure. **b** The bias of complex BN after learning process. **c**, **d** CD compensation taps in frequency domain after learning process. **e**–**h** 2 × 2 MIMO taps in frequency domain after learning

To investigate the factors contributing to the performance gain, this section focuses on the learned parameters obtained after the learning process. The analyses focus on the single-channel transmission over 1600 km, and similar conclusions are applicable to WDM transmissions. Figure 5a displays the LOFO estimations throughout the learning process. It is observed that LDSP achieves convergence after step 1, which involves CMA training. The LOFO values remain stable during the MMA training and MSE tracking processes, indicating the slow-varying nature of LOFO.

Figure 5b showcases the bias parameters of the complex BN module. The bias parameters are presented in the frequency domain, revealing several peak values. It is important to note that due to the sub-optimal nature of DA and AD conversion, clock information may be introduced into the signal, potentially leading to a degradation in signal performance. However, the biases in the BN module can be utilized for compensating clock leakage effects. Moreover, since bias operations involve simple addition operations that can be easily executed in application-specific integrated circuits (ASIC), the computational complexity associated with biases is not significant. The utilization of the learning algorithm in LDSP enhances the capabilities of DSP systems, enabling them to possess enhanced functionalities and adaptability.

In Fig. 5c, d, the CD taps for the X and Y polarizations of the signal are depicted, respectively. The amplitude response of the taps represents an all-pass filter, while the phase response reflects the CD effects. In Fig. 5e–h, the learned 2 × 2 MIMO taps are displayed. The amplitude response exhibits an M-shaped filter characteristic, which can be attributed to the system’s non-ideal system response. Additionally, the MIMO taps compensate for residual CD even after CD compensation, resulting in a phase response that aligns with the CD compensation. Importantly, these responses align with those observed in traditional DSP, highlighting the high interpretability of LDSP and its consistency with theoretical expectations.

**Fig. 6:**
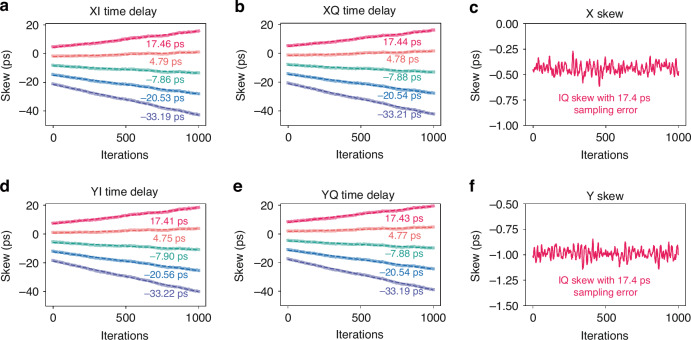
**The learned skew of LDSP.** **a**, **b** Learned XI and XQ time delay with different sampling errors. **c** IQ skew between the XI and XQ time delay with 17.4 ps sampling error. **d**, **e** Learned YI and YQ time delay with different sampling errors. **f** IQ skew between the YI and YQ time delay with 17.4 ps sampling error

Figure 6 illustrates the learned skew in the LDSP framework. Notably, Fig. 6c, f demonstrates the tracking of skew changes, highlighting the robustness of LDSP in handling IQ skew, where the skew of X and Y branch converge to −0.5 and −1.0 ps, respectively. Traditional DSP methods encounter difficulties when it comes to compensating for IQ skew without introducing additional complexity. To address IQ skew, techniques such as 4 × 2 MIMO or widely linear MIMO are often employed. However, these techniques come with increased power burdens and additional computational requirements. It is important to note that the LDSP framework does not include time recovery. Instead, LDSP is capable of detecting and adjusting timing errors, as evidenced by the results shown in Fig. 6a, b, d, e, where the time delay exhibits a linear function at each branch. If we divide the time series into several blocks as shown in Fig. 2., where time space of each sample is Δ*t*, then the whole time series of an IQ branch can be represented by:

4 $$x(t)=\sum _{r}{x}_{r}(t-rR\Delta t)$$

where *x*_*r*_ is the *r*-th block, *R* is the sample number in each block. If we use *τ*_*r*_ to compensate IQ skew at *r*-th block with the sampling error, then the compensation procedure in frequency domain can be presented by:

5 $$Y({\varOmega }_{k})=\sum _{r}{X}_{r}({\varOmega }_{k})\exp (-j{\varOmega }_{k}rR\Delta t)\exp (-j{\varOmega {^\prime} }_{k}{\tau }_{r})$$

where ${\varOmega }_{k}=2{\pi }{k}{/}{R}{\Delta }{t}$ reflecting the digital frequency and ${\varOmega }_{{\rm{k}}}^{\prime}$ is the digital frequency with biased sampling rate. To reduce the sampling rate error, the compensated signals should be:

6 $$Y({\varOmega }_{k})=\sum _{r}{X}_{r}({\varOmega }_{k})\exp (-j{\varOmega {^\prime} }_{k}(rR\Delta t+b))$$

where *b* is a constant value. It is necessary to make Eq. (5) and Eq. (6) equal, that is, for *τ*_*r*_, it satisfies:

7 $${\tau }_{r}=(\Delta t-\Delta t^{\prime} )Rr+b$$

where ${\Delta}t-{\Delta}{t}^{\prime}$ is a slope of *τ*_*r*_, representing the time sampling error. The slopes of IQ skew during the processing represent the digital sampling errors, while the bias of IQ skew means the time delay. In this way, LDSP can find and compensate the timing error using the IQ skew compensation module. In Fig. 6a, b, d, e, we estimate several slopes by manually adjusting the sampling error from −33.2 ps to 17.4 ps before DSP. The values depicted in these figures represent the estimated slopes at a 1.25× oversampling rate, calculated according to Eq. (7). These estimates demonstrate that the slopes are consistent with the corresponding sampling errors. Notably, the time error remains consistent across XI, XQ, YI, and YQ channels, owing to their shared sampling clock. Despite this, we observe minor slope discrepancies, within a ±0.05 ps range, for the same sampling error. This variability in slopes across different sampling rate errors is successfully detected by LDSP. By using the several block of learned time delay values of LDSP, we can estimate the timing error and build feedback to control the sampling rate. Importantly, LDSP achieves this without the need for a separate time recovery module, thereby optimizing the utilization of DSP resources and showcasing its high efficiency and robust signal compensation abilities.

### Complexity analysis

It is well known that the processing of samples per symbol determines the DSP complexity. In this part, we test different processing rate, from 1.0 to 2.0 samples/symbol. Note that due to the sample size changes with different processing rate, the dynamic effects are also changed, indicating that the LR need to be adjusted. Typically, when using lower processing rate, one can use higher LR to track the faster dynamic effects.

**Fig. 7:**
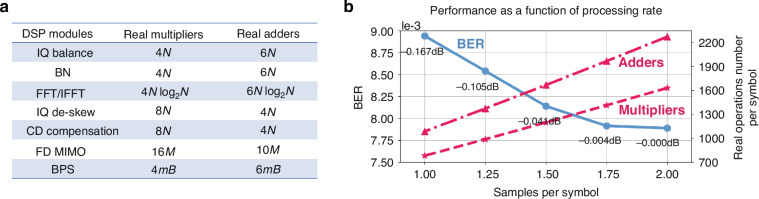
**The complexity of LDSP framework.** **a** The complexity of the DSP modules. **b** The performance as a function of processing rate. The left axis represents the BER changes while left axis showing the complexity tendency

In order to determine the complexity of LDSP, it is necessary to establish the definitions for block samples and symbols. Let us denote the sampling rate, processed block symbols, and mini-block symbols as *r*, *n*, and *m*, respectively. Moreover, the block and mini-block samples can be represented as *N* and *M*, where *N* is equal to 2*rn* and *M* is equal to 2*rm*, considering the overlap-and-save methods. The BPS test phase number is denoted as *B*. Subsequently, the concrete representation of real multipliers and adders can be observed in Fig. 7a. Assuming that a block comprises *k* mini blocks, the complexity of the multipliers and adders can be expressed as follows:

8 $$\begin{array}{c}{\rm{Multipliers}}=\frac{8N{\log }_{2}(N)+28N+k[8M{\log }_{2}(M)+16M+4mB]}{n}\\ {}{\rm{Adders}}=\frac{12N{\log }_{2}(N)+34N+k[12M{\log }_{2}(M)+10M+6mB]}{n}\end{array}$$

The utilization of the error backpropagation approach introduces an additional complexity, doubling the complexity depicted in Eq. (8). Within the context of this study, the block symbol is set at 512, while the mini block symbol is defined as 64. The BPS employs 8 test phases. By incorporating the sample rate and symbol number into Eq. (9), the complexity and performance outcomes, as a function of the processing rate, are presented in Fig. 7b.

The left axis of Fig. 7b illustrates the BER performance for single-channel 1600 km transmission with varying sampling rates. It can be observed that an increased processing rate correlates with an improved BER performance. Nonetheless, the improvements achieved by elevating the processing rate are relatively marginal, suggesting that LDSP exhibits a limited sensitivity to changes in the sampling rate.

The Q factor and BER performance loss is marked compared to 2 samples per symbol in Fig. 7b. LDSP demonstrates remarkable robustness in BER even when operated at the symbol rate, as evidenced by a minimal decrease of less than 0.17 dB in Q factor. Notably, the complexity of LDSP can be diminished by reducing the processing rate, as illustrated on the right axis of Fig. 7b. Processing at the symbol rate leads to a substantial 48% decrease in complexity compared to a processing rate of 2 samples per symbol, reducing the number of multipliers from 2272 to 1088. This emphasizes the cost-effectiveness of the LDSP learning framework, which enables optimal performance while keeping complexity within manageable limits.

## Discussion

This paper presents an experimental demonstration of the significant performance improvements and computational savings achieved by integrating traditional DSP algorithms into a DL framework. The comparative analysis of performance is conducted post-optimization of hyperparameters for the traditional DSP. By leveraging DL optimization techniques, DSP algorithms can effectively compensate for various channel distortions, including clock leakage, skew tracking, and even time recovery, with minimal additional complexity. The optimized parameter configurations obtained through DL optimization also provide valuable insights into the linear effects in the transmission system. LDSP retains the traditional block-design DSP structure while utilizing gradients as inherent feedback paths, facilitating interconnections among DSP modules, and leading to higher performance gains. The learnable framework of LDSP enables optimal performance within limited complexity constraints. Notably, the LDSP module achieves symbol-rate processing with negligible performance costs. Due to the DL framework, the LDSP is compatible with DL structures and can be extended to incorporate learnable perspectives for nonlinear compensation in the future. The LDSP could emerge as a new and highly efficient benchmark for linearity compensation, generating significant interest across various domains of nonlinear compensations and beyond.

## Materials and methods

### Experiments setup

The experiment setup is shown in Fig. 2a. At the transmitter side, an 80 GSa/s arbitrary waveform generator (AWG) with 17 GHz bandwidth is used to generate 50-GBaud 16-QAM symbols shaped by squared raised cosine filter with roll-off factor of 0.1. The electrical waveforms first go through a 40 GHz bandwidth coherent driver modulator (CDM) to modulate the optical signals. The modulated optical waveform is amplified using erbium-doped fiber amplifiers (EDFA) with 5.0 dB noise figure and launched into the fiber link. The fiber laser with linewidth around 100 kHz is used at both transmitter and receiver side. An EDFA module is used following with the fiber transmission to compensate for the fiber loss incurred over all the spans. The fiber spans approximately 80 km, and a single-channel transmission utilizes a series of 20 spans, totaling 1600 km. In wavelength division multiplexing (WDM) long-haul transmission, the channel under test (CUT) is produced by the AWG. Concurrently, additional interference channels originate from amplified spontaneous emission (ASE) noises. The CUT is centrally located among 21 WDM channels, traversing a 1600 km optical fiber link in this work. After fiber link transmission, the optical waveforms are coherently detected using 40-GHz bandwidth integrated coherent receiver (ICR). And the signals are sampled by a 100 GSa/s digital oscilloscope with 30-GHz electrical bandwidth.

### Perturbation nonlinear compensation

The PNC employed in this paper is originated from the first-order perturbation of the nonlinear Schrödinger equation. When complete CD is compensated by the receiver DSP, the nonlinear perturbation terms of *X* polarization can be represented by^17–19,40^:

9 $$\Delta {A}_{X}=\sum _{m,n}{P}_{0}^{3/2}({A}_{n,X}{A}_{m+n}^{\ast }{A}_{m,X}+{A}_{n,Y}{A}_{m+n,Y}^{\ast }{A}_{m,X}){C}_{M,N}(m,n)$$

where *A*_*n*,*X*_ and *A*_*n*,*Y*_ represent the transmitted symbols for *X* and *Y* polarizations, respectively, indexed by *n*. The *P*_0_ denotes the launch power, and *C*_*M*,*N*_(*m*, *n*) is the matrix of first-order perturbation coefficients, where the size is *M*$\times$*N*, which can be optimized with respect to the link parameters. The nonlinear perturbation terms for *Y* polarization can be obtained from Eq. (9), substituting *X* indices with *Y* indices.

To compensate for the nonlinear interactions using PNC in the receiver side, it is necessary to pre-determine the products of three symbols. In this paper, we replace the transmitted symbols by the received symbols after DSP and decision. The coefficient matrix is optimized via machine learning techniques, utilizing a training set of known symbols and the given link parameters. Following the computation of perturbation terms as Eq. (9), PNC effectively removes nonlinear impairments by:

10 $${\hat{A}}_{X}={\tilde{A}}_{X}-\Delta {A}_{X}$$

where the ${\hat{A}}_{X}$ and ${\tilde{A}}_{X}$ are PNC output and input symbols, respectively.

**Fig. 8:**
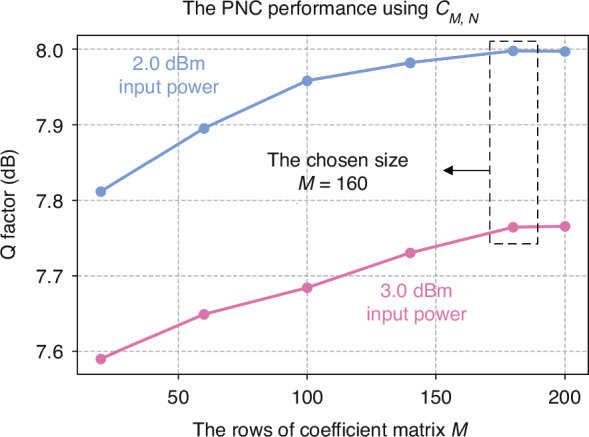
**The PNC performance with varying coefficient matrix sizes.** The 2.0 and 3.0 input power single-channel signals are investigated over 1600 km distance. The coefficient matrices used in the PNC are square, and the horizontal axis of the graph displays the number of rows in each matrix

The size of the coefficient matrix for PNC significantly influences both the effectiveness of the compensation and the system complexity. Essentially, the matrix size indicates the capacity to capture the time correlation of nonlinearities within the system. Thus, enlarging the coefficient matrix size tends to enhance nonlinear compensation performance, until it sufficiently encompasses the majority of nonlinear time correlations. Beyond this point, further increases in matrix size do not yield performance improvements. The testing of PNC performance with varying matrix sizes, as depicted in the Fig. 8, provides valuable insights for optimizing the PNC system, particularly in single-channel 1600 km transmission scenarios. The results indicate that the optimal number of rows in the matrix for this specific transmission length is 160. Beyond this point, increasing the matrix size does not yield additional performance improvements.
